# Effect of Mo Addition on the Susceptibility of Advanced High Strength Steels to Liquid Metal Embrittlement

**DOI:** 10.3390/ma18061291

**Published:** 2025-03-14

**Authors:** Fateme Abdiyan, Joseph R. McDermid, Fernando Okigami, Bita Pourbahari, Andrew Macwan, Mirnaly Saenz de Miera, Brian Langelier, Gabriel A. Arcuri, Hatem S. Zurob

**Affiliations:** 1 Department of Materials Science and Engineering, McMaster University, Hamilton, ON L8S 4L8, Canada; 2 Hexagon Manufacturing Intelligence, 46444 Hexagon Way, Novi, MI 48377, USA; 3 ArcelorMittal Dofasco, 1330 Burlington Street East, Hamilton, ON L8N 3J5, Canada; 4 Canadian Center for Electron Microscopy, 1280 Main Street West, Hamilton, ON L8S 4L8, Canada

**Keywords:** liquid metal embrittlement, atom probe tomography, electron microscopy, resistance spot welding

## Abstract

Liquid metal embrittlement (LME) in Zn-coated advanced high-strength steels (AHSSs) is an increasing concern, particularly in automotive assembly, where it can cause early failure and reduce ductility during resistance spot welding (RSW). This study explores the impact of adding 0.2 wt% Mo on the LME susceptibility of 0.2C-2Mn-1.5Si AHSS through hot tensile testing, RSW, and advanced microstructural analyses, including atom probe tomography (APT) and transmission electron microscopy (TEM). The results suggest that Mo enhances resistance to LME, as evidenced by the increased tensile stroke from 2 mm in the case of the 0 Mo alloy and to 2.75 mm in the case of the 0.2 Mo sample. Also, the average crack length in the shoulder of the welded samples decreased from 109 ± 7 μm to 28 ± 3 μm by adding 0.2 wt% Mo to the base alloy. APT analysis revealed that, in the presence of Mo, there is increased boron (B) segregation at austenite grain boundaries, improving cohesion, while TEM suggested more diffusion of Zn into the substrate, facilitating the formation of Zn-ferrite. These findings highlight Mo’s potential to reduce LME susceptibility of AHSS for automotive applications.

## 1. Introduction

Liquid Metal Embrittlement (LME) is a materials degradation phenomenon whereby a normally ductile metal becomes brittle and fails prematurely when exposed to an aggressive liquid metal under an applied stress, usually via intergranular fracture [1]. Automotive structures currently employ large fractions of advanced high strength steels (AHSSs), the majority of which are cathodically protected against aqueous corrosion using Zn-based metallic coating systems [2]. This increases the risk of LME when high-temperature procedures such as welding and direct hot stamping are involved [3]. LME is of particular concern during resistance spot welding (RSW), the dominant joining technology used in the automotive industry [4]. Studies have shown that LME cracks can severely compromise the performance of welded joints, leading to their premature failure [5]. The growing use of AHSSs and ultra-high strength steels (UHSSs) in the automotive industry means that LME poses a significant challenge for future vehicle designs, particularly as the industry moves to lighter body-in-white designs to increase fuel efficiency and increase electric vehicle battery range without compromising structural integrity [6].

Molybdenum (Mo) is a key alloying element in steels due to its ability to significantly enhance high-temperature strength and toughness [7,8,9], and steel hardenability [10]. Also, Mo forms stable carbides, which can further enhance steel mechanical properties [11]. Additionally, by segregating on grain boundaries, Mo is reported to improve grain boundary (GB) cohesion, thereby increasing resistance to intergranular cracking, temper and hydrogen embrittlement [12,13,14,15].

Despite significant progress in understanding LME and the role of Mo in mitigating various forms of embrittlement, there remains a knowledge gap with respect to how Mo influences AHSS LME susceptibility. In particular, the effects of Mo on the mechanical properties and LME susceptibility of steels in the presence of other alloying elements such as Mn and Si have not been fully explored. Thus, the objective of the current research is to determine the mechanism by which Mo additions to AHSSs can affect LME susceptibility.

## 2. Materials and Methods

In this study, the experimental materials consisted of two electro-galvanized advanced high-strength steels (AHSS) with chemistries (Table 1) differing only in their Mo contents. As per Table 1, the experimental alloys will be referred to as 0 Mo and 0.2 Mo in the subsequent text. A quench and partitioning (Q&P) microstructure, essential for advanced mechanical properties, was developed through salt bath heat treatments. The samples were first austenitized at 900 °C for 120 s, followed by isothermal holding at 350 °C for 300 s.

To investigate the effect of Mo addition during resistance spot welding (RSW), Zn coated specimens were welded using a standard dome-shaped copper electrode (6 mm tip diameter). Zn coating was applied on the steel sheets using electro galvanizing (EG) to a coating thickness of 10–12 μm. The RSW parameters were carefully controlled, with a welding time of 383 milliseconds, a clamping force of 4.5 kN, and a maximum welding current between 11 and 12 kA. The welding conditions and chemical composition of the 0.2 Mo sample were then used to simulate the time–temperature (t-T) profile experienced by the sample during RSW.

**Figure 1:**
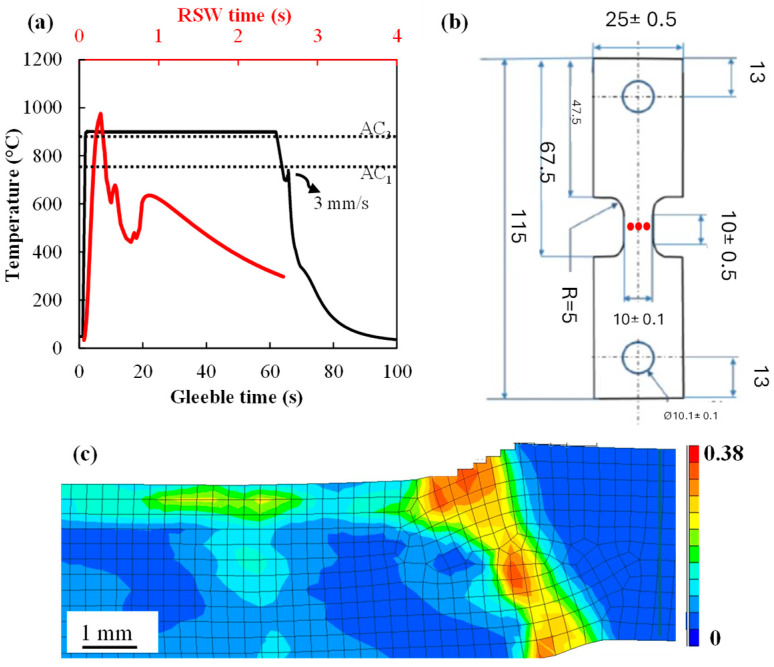
(**a**) The time–temperature profile of a resistance spot weld obtained through Simufact simulations (red curve) and the time–temperature design for Gleeble experiments (black curve), (**b**) geometry of Gleeble tensile coupons (in mm) along with the location of thermocouples, shown as red dots, and (**c**) equivalent plastic strain simulations for sample undergoing RSW.

To compare the relative LME susceptibly of the 0 and 0.2 Mo alloys during hot tensile testing, tensile tests were carried out using a Gleeble 3500 thermomechanical simulator (Gleeble, Poestenkill, NY, USA). The time–temperature profile for the Gleeble experiments was designed based on the t-T profile of the shoulder region during welding that was simulated using Simufact software 2024.1 [16]. The simulations provided useful insight into the thermal profile and strain field generated in the material in the shoulder region during RSW, as depicted in Figure 1a. The simulation process is detailed in Appendix A. As can be seen in Figure 1a, the RSW sample experienced a maximum temperature higher than the austenization temperature (Ac_3_), which was determined to be 892 °C using dilatometry. But, due to the high heating rate (1000 °C/s) of RSW and short exposure time to the temperatures above Ac_3_, it was a matter of speculation whether full austenization occurred in the shoulder. Therefore, the thermal cycle of hot tensile tests was designed with a different time scale to ensure austenization was completed during the Gleeble experiments.

Therefore, Gleeble thermo-mechanical testing comprised heating at 1000 °C/s to 900 °C, holding at 900 °C for 60 s, cooling at 100 °C/s to 700 °C and deforming immediately to fracture using a 3 mm/s cross-head speed. Gleeble tests were carried out on tensile coupons with the gauge length electrogalvanized with a pure Zn coating of approximately 10 μm thickness. The Gleeble specimen geometry is depicted in Figure 1b. The uncoated side of the Gleeble gauge length had three K-type thermocouples welded at the locations specified by the red dots in Figure 1b for temperature control and monitoring [17].

After RSW and hot tensile testing, the surfaces and cross-sections of the specimens were observed under a light optical microscope and electron microscope to compare the severity of LME cracking in fractured tensile samples and the shoulder region of RSW samples. The reason for focusing on shoulder region is that this area experiences relatively high strain (Figure 1c) and LME cracks are usually observed in this region of a resistance spot weld.

For the characterization of the Gleeble and RSW samples, cross-sections were cold mounted in epoxy resin to preserve their structural integrity. Samples were polished to a mirror finish using standard metallographic techniques and a thin conductive carbon coating (~10 nm) was applied to the polished surface to minimize sample charging while examining them via scanning electron microscopy (SEM) and electron backscattered diffraction (EBSD). SEM and EBSD analyses were conducted using a Thermo Fisher Scientific (Waltham, MA, USA) Apreo 2 S LoVac high resolution field emission scanning electron microscope (FEG-SEM) equipped with a Symmetry 3 EBSD detector. The operating parameters included an accelerating voltage of 20 kV and a spot size of 0.08 µm, ensuring high-resolution crystallographic mapping. The reconstruction of prior austenite grains from the EBSD data was performed using OR-tools, an open-source MATLAB R2019a toolbox [18].

To investigate the elemental distribution within the LME cracks at a finer scale, a Thermo Fisher Scientific Helios 5 UXe DualBeam Plasma-Focused Ion Beam SEM (FIB-SEM) was used to extract thin lamellae for scanning transmission electron microscopy (STEM) coupled with energy-dispersive X-ray spectroscopy (EDS). Before FIB milling, protective layers of tungsten and carbon were deposited on the regions of interest to prevent ion beam-induced damage. STEM micrographs and STEM-EDS spectra were acquired using a Thermo Fisher Scientific Talos F200X transmission electron microscope operating at 200 kV and a spot size of 0.7 nm, allowing high-resolution imaging and chemical analysis at the nanoscale.

In addition, atom probe tomography (APT) was utilized to determine the local composition at the atomic scale, focusing on LME crack tips. FIB-SEM was used to prepare APT needles from specific regions of interest, typically located along LME cracks or prior austenite grain boundaries (PAGBs) near LME crack tips. APT analysis was performed using a Cameca LEAP 5000 XS (Madison, WI, USA) system, operating at 50 K with a laser pulse of 60 pJ. The resulting data were reconstructed into 3D atomic maps using AP Suite 6 and IVAS 3.8.12 software.

This combined approach of advanced characterization across several magnitudes of length scales and simulation allowed for a thorough investigation of the effects of Mo content on the LME susceptibility of resistance spot-welded AHSS from material to process.

## 3. Results

### 3.1. Mechanical Testing

**Figure 2:**
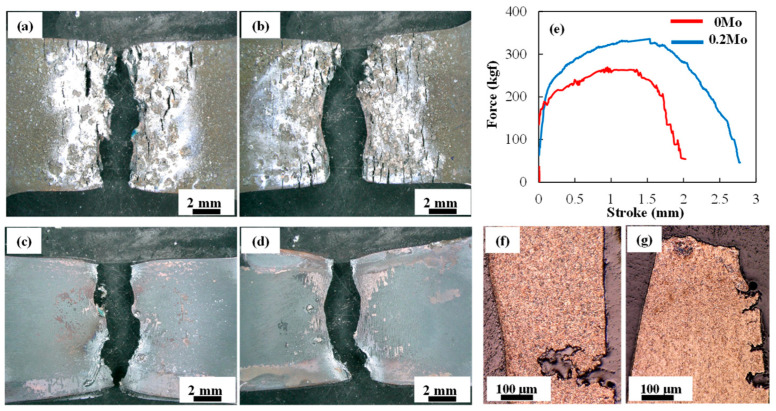
Zn-coated side of tensile coupons after fracture for (**a**) 0 Mo and, (**b**) 0.2 Mo alloy, bare side of fractured tensile coupons for (**c**) 0 Mo and (**d**) 0.2 Mo alloy, (**e**) force–stroke diagram of the samples from hot tensile testing, and cross-section of tensile samples showing LME cracks in (**f**) 0 Mo, and (**g**) 0.2 Mo sample.

Typical results from the hot tensile testing of Zn-coated 0 Mo and 0.2 Mo samples are given in Figure 2. All cracking occurred on the Zn-coated side of the tensile coupons, as observed when comparing their bare and Zn-coated sides (i.e., Figure 2a vs. Figure 2c for the 0 Mo sample, and Figure 2b vs. Figure 2d for the 0.2 Mo sample). The force-stroke diagrams derived from the hot tensile tests are displayed in Figure 2e and indicate that the 0.2 Mo sample exhibits both higher force and stroke, suggesting that it is more resistant to LME cracking. Figure 2f,g shows the cross-sections of the tensile samples, where the Zn-coated side is on the right and the bare side on the left, showing that LME cracks originated on the Zn-coated side and propagated toward the bare side for both alloys.

It is clear that LME cracking in the 0 Mo sample (Figure 2a,f) is more severe because of deeper penetration, i.e., longer cracks, and more crack openings, i.e., higher crack width. The Gleeble stroke, which is proportional to the ductility of the material, has an inverse relationship with the severity of cracking.

**Figure 3:**
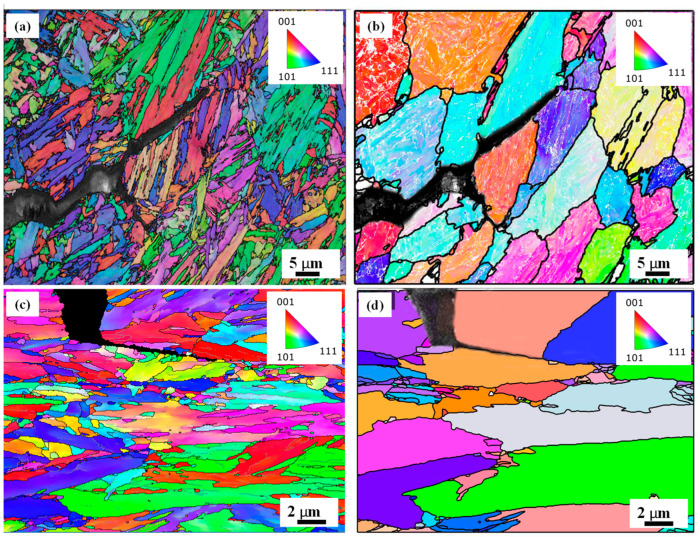
(**a**) IPF-EBSD map, (**b**) PAG reconstruction of a region containing LME crack tip in the cross section of 0 Mo hot tensile sample, and (**c**,**d**) show the same maps for the 0.2 Mo sample.

Figure 3a,b shows the EBSD results for a region containing an LME crack tip in the cross-section of the Gleeble 0 Mo sample, and Figure 3c,d shows the same for the 0.2 Mo alloy, in which (a) and (c) display the inverse pole figure (IPF) maps, and (b) and (d) show the reconstructed prior austenite grain (PAG) maps. The reconstructions in (b, d) show that the LME crack propagated along PAGBs, which are known to be more susceptible to LME [19]. This observation showed that austenite grain boundaries, which were freshly formed after holding above Ac_3_ and full austenization, were susceptible to LME cracking during the deformation. 

### 3.2. Resistance Spot Welding

**Figure 4:**
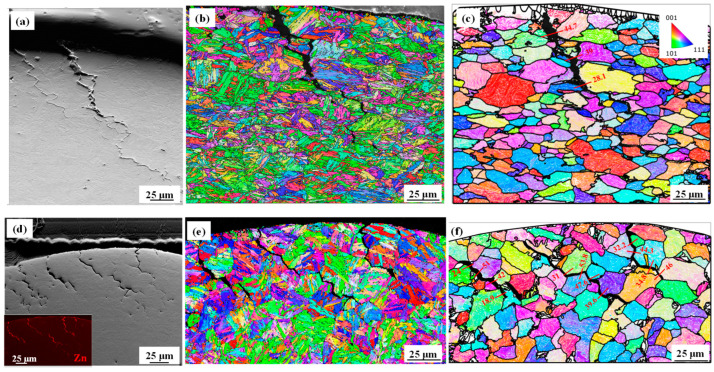
(**a**) SEM image, (**b**) EBSD map, and (**c**) the reconstructed PAGs for the 0 Mo sample; (**d**) SEM micrograph, (**e**) EBSD map and (**f**) PAG reconstruction for the 0.2 Mo welded sample.

The microstructural characterization of the weld shoulder for the 0 Mo and 0.2 Mo resistance spot-welded samples is depicted in Figure 4. The SEM images in Figure 4a,d compare the crack length for the 0 Mo and 0.2 Mo, respectively, and the EDS insert of 0.2 Mo sample in Figure 4d indicates that Zn has filled the LME cracks, as indicated by the red color. The IPF maps obtained in the cracked areas are displayed in Figure 4b,e. According to the PAG reconstructions in Figure 4c,f, the LME cracks propagate along PAGBs with misorientation angles ranging from 15° to 55° in both alloys. The similarity of these results with those of the Gleeble experiments indicates that austenization occurred during RSW too, and, similar to the Gleeble samples, the RSW shoulder region had also contained austenite grains at the temperatures of LME cracking. This observation, along with microhardness values similar to the Gleeble samples (~545 ± 8 Hv), indicates that the PAGBs seen in Figure 4 are the postmortem observations of AGBs present during the welding process.

**Figure 5:**
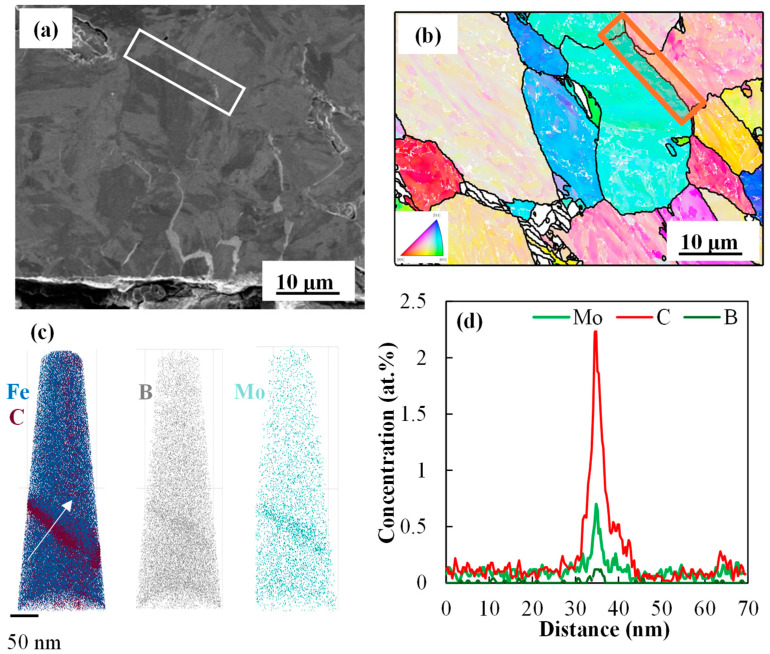
(**a**) FIB image of a cracked area in shoulder region of the RSW 0.2 Mo alloy, (**b**) PAGs reconstruction marking the PAGB of interest using a red rectangle (same boundary depicted in “(**a**)” using a white rectangle for visibility), (**c**) the 3D APT tip reconstructions, and (**d**) the 1D elemental profiles of C, B, and Mo along the white arrow in (**c**).

A FIB-SEM image of the 0.2 Mo RSW sample shoulder region is shown in Figure 5a, where the LME cracks are visible as brighter areas as they are Zn-alloy filled. As can be seen in Figure 5a,b, a prior austenite grain boundary near the LME crack tip was chosen that was not yet cracked and which experienced similar thermal cycles with the cracked grain boundaries. This region was also chosen as it was almost perpendicular to the tensile strain applied to the shoulder [20]. Figure 5b displays the prior austenite grains reconstructed from the IPF-EBSD map of the region, which marks the PAGB that was subjected to APT analysis. The elemental distribution (along the white arrow in the 3D APT reconstructions in (c)) is seen in Figure 5d. According to Figure 5d, it is observed that C, B, and Mo are co-segregating on the PAGB.

**Figure 6:**
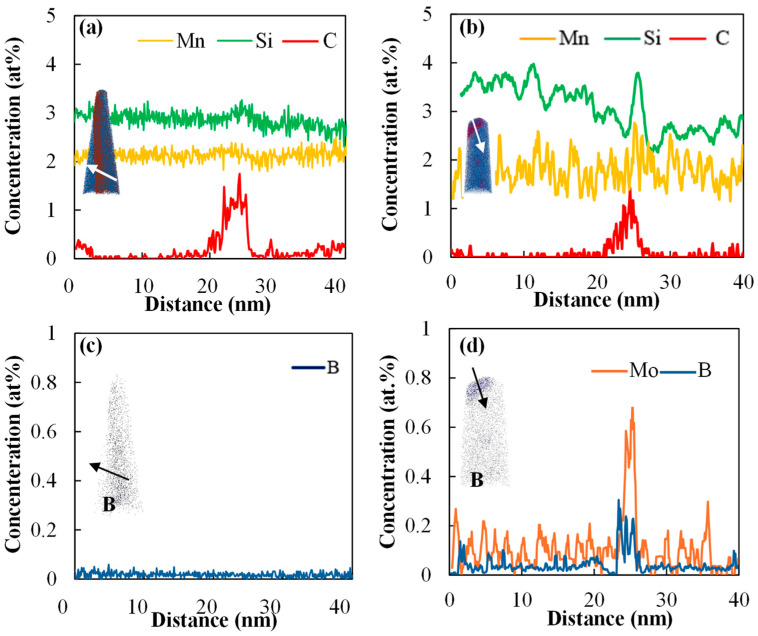
APT results for the 0 Mo (**a**,**c**) and 0.2 Mo sample (**b,d**) of a PAGB close to a LME crack tip in which (**a**,**b**) show the 1D profile for C, Si, and Mn, (**c**) displays the 1D profile of B for the 0 Mo sample, and (**d**) the 1D profile of Mo and B for the 0.2 Mo sample along the arrow.

For the 0 Mo RSW sample, Figure 6a,c displays the APT data from a prior austenite grain boundary adjacent to an LME crack tip, where Figure 6b,d displays the same information for the 0.2 Mo sample. The C, Si, and Mn profiles at the boundaries are comparable (as shown in Figure 6a,b); however, Figure 6c,d indicate that B segregation differs and a higher PAGB B concentration was observed in the case of the 0.2 Mo sample.

**Figure 7:**
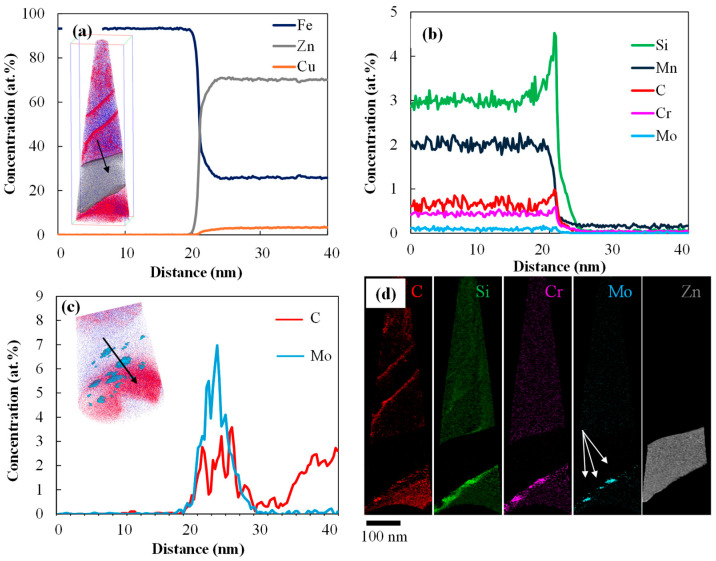
APT results for the 0.2 Mo sample containing a LME crack in which (**a**) shows the 1D elemental profile for Fe, Zn, and Cu, (**b**) the 1D elemental profile of Si, Mn, C, Cr, and Mo, (**c**) 1D elemental profile along Mo-rich carbides, and (**d**) the corresponding 2D reconstructed maps pointing at the Mo-rich carbides using white arrows.

The APT results from a crack in the 0.2 Mo RSW sample are shown in Figure 7. 1D elemental profiles along the black arrow in (a) are seen in Figure 7a,b. According to Figure 7a, the material inside the LME crack has a composition of approximately 70 at% Zn, 25 at% Fe, and 4 at% Cu, indicative of Fe_3_Zn_7_ with some dissolved Cu, likely originating from the welding electrodes. With extremely restricted solubility in the Zn liquid, C and Si are rejected to the Fe side of the solid-liquid interface, as seen in Figure 7b. Since Mn has the highest solubility amongst all of these minor alloying elements in liquid Zn [21], it is the only alloying element that is not entirely rejected from the liquid Zn.

The 1D profiles of C and Mo along the Mo-rich carbides that are marked with arrows in the reconstructed APT maps in Figure 7c,d, are displayed in Figure 7c. The blue ellipsoids in Figure 7c are Mo-rich carbides, along which their 1D elemental profiles are extracted. These particles contain Mo and C with a 7:3 ratio, and ThermoCalc 2023b calculations show that M_7_C_3_ carbides form inside the steel matrix at 600–700 °C. Therefore, these particles are speculated to be M_7_C_3_.

**Figure 8:**
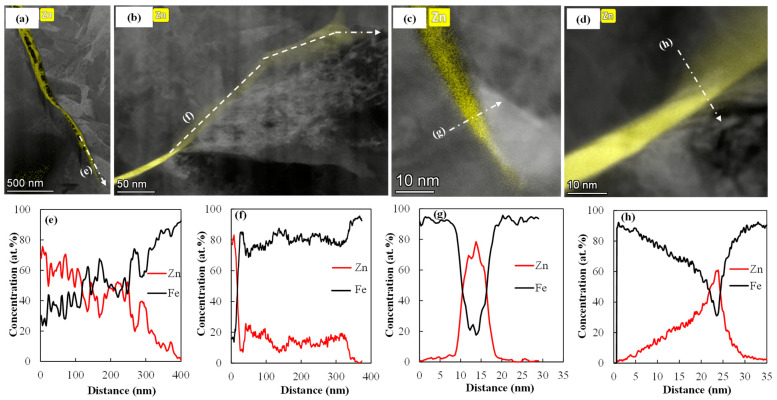
(**a**,**c**) STEM-EDS maps of LME cracks in the shoulder of the 0 Mo and (**b**,**d**) 0.2 Mo RSW samples with Zn distribution in yellow, (**e**,**f**) Fe and Zn profiles along the crack length and (**g**,**h**) Fe and Zn profiles normal to the crack for 0 Mo and 0.2 Mo, respectively, locations of which are displayed using the white dashed arrows in the top images and labelled the same as the corresponding line scans below.

The Zn distribution maps of LME shoulder cracks in 0 Mo and 0.2 Mo RSW samples are superimposed over the HAADF STEM images in Figure 8a,b, respectively, where the white arrows indicate the location of the line scans. Figure 8e,f shows the corresponding Zn and Fe profiles along the crack according to the previously described white arrows. In the 0 Mo alloy, Zn concentration gradually drops along the crack as seen in Figure 8e, without any indication of a diffusion zone either ahead of or close to the LME crack. The 0.2 Mo alloy exhibits a high Zn concentration inside the LME crack, followed by a diffusion zone ahead of the LME crack that contains approximately 15 at% Zn, as seen in Figure 8f. The presence of a layer of approximately 200 nm ahead of crack tip, which contains ~15 at% Zn, implies that the diffusion of Zn into austenite has passed the solubility limit of austenite for Zn, resulting in the formation of Zn-ferrite (α Fe(Zn)) along this boundary. Figure 8g,h demonstrates the concentration profiles of Fe and Zn along the arrows normal to the LME crack tip for the 0 Mo and 0.2 Mo alloys, respectively, as shown in Figure 8c,d. The Zn profile for the 0 Mo alloy depicted in Figure 8g shows a high concentration of Zn inside the LME crack with a sharp drop to zero, indicative of the presence of liquid Zn without the occurrence of a reaction with the substrate, whereas the 0.2 Mo sample Zn profile in Figure 8h shows lateral diffusion of Zn into the steel substrate and the formation of Zn-Fe reaction products.

## 4. Discussion

The results of RSW simulation in Figure 1a (the red curve) show that the shoulder region of the weld experiences a maximum temperature in excess of the Ac_3_ during the RSW process, but, due to the high heating rate and limited time cycles of RSW, Gleeble experiments were designed to determine whether austenization occurs during RSW.

Figure 2 illustrates that the addition of 0.2 wt% Mo to the Zn-coated specimens enhances mechanical performance, as evidenced by increased stroke, and reduces susceptibility to liquid metal embrittlement (LME). Grain refinement was observed using ImageJ 1.45 [22], with the average grain size decreasing from 14 ± 2 μm to 10 ± 1 μm upon the addition of 0.2 wt% Mo to the base advanced high-strength steel (AHSS). This finding aligns with prior studies, such as Jo et al. [23], who reported that the solute drag effect of Mo contributes to grain refinement in hot-stamping steels. Figure 2 also shows less severe cracking in the cross section of the 0.2 Mo Gleeble sample. The beneficial effect of grain refinement in reducing embrittlement in steel is reported in the literature [24]; however, since the difference in the grain sizes in current study is not significant, another reason must have been responsible for the different LME susceptibilities.

Figure 3a,c presents inverse pole figure (IPF) maps for tensile-tested 0 Mo and 0.2 Mo samples, respectively, alongside their prior austenite grain (PAG) reconstruction maps in Figure 3b,d. It is observed that the LME cracks propagate preferentially along austenite grain boundaries, which are the favored paths for LME crack propagation, as corroborated by Akbari et al. [25] due to their low fracture strength and being sensitive sites for atomic decohesion [26].

Figure 4 shows the LME crack propagation along the PAGBs for the LME cracks inside the shoulder of the welded samples. The similarity between the microstructure and microhardness values of the Gleeble and welded samples confirm that the microstructure of both samples was similar at the process temperature. Therefore, similar to Gleeble experiments, new austenite gains were formed during the RSW process, and the post-mortem analysis of PAGBs at room temperature reveals the microevents occurring along the austenite grain boundaries at the welding temperatures.

Specifically, Figure 4c,f shows that LME cracks propagate along the PAGBs with a high misorientation angle. It is also reported in the literature that GBs with high misorientation angles, especially those higher than 15°, are less stable than those with low angle [27]. Bhattacharya et al. [28] also reported Zn infiltration along the austenite grain boundaries, due to the high energy of the austenite GBs with high angle misorientation, making them prone to intergranular fracture [29]. This figure also shows that, compared to the 0.2 Mo alloy, the LME cracks in the 0 Mo alloy propagate further into the cross-section. Overall, the 0.2 Mo sample shows a lesser crack severity index:

(1) $$CI=\frac{nL}{t}$$

in which *n* is the number of cracks, *L* is the lognormal median crack length, and *t* is the sheet thickness [19]. Using Equation (1), the *CI* values for the 0 Mo and 0.2 Mo alloys were 0.61 and 0.45, respectively.

The co-segregation of B, Mo, and C along the PAGB is noted, per Figure 5. Yoo et al. [14] also highlighted the tendency of C, B, and Mo for co-segregation at prior austenite grain boundaries. Li et al. [30] also noted the segregation of C, B, and Mo at the PAGBs. Similar to the current study, they observed C and Mo enrichment at PAGBs that were five–eight times higher than their bulk concentration.

**Figure 9:**
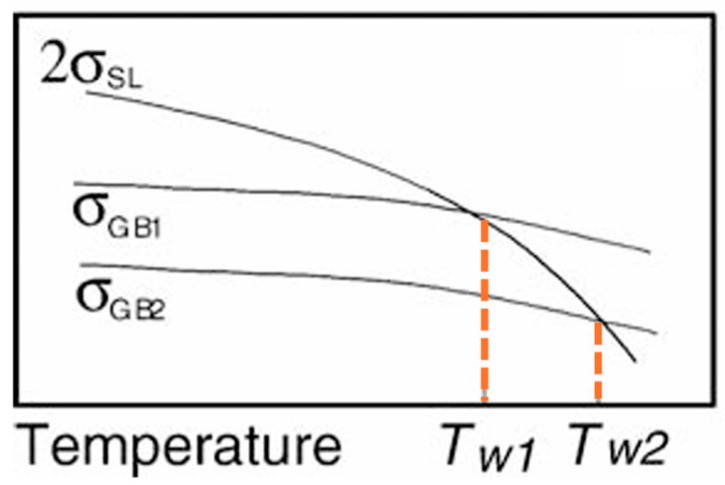
Scheme of the wetting temperature dependence on the GB energy, in which σ_GB_ is the GB energy and σ_SL_ is the energy of the solid–liquid interface [34].

Figure 6 demonstrates pronounced B segregation along PAGBs in the 0.2 Mo alloy compared to the 0 Mo alloy, meaning that Mo has enhanced B segregation on the PAGB. This observation is consistent with the literature. Dumbill et al. [31] attributed this phenomenon to the strong binding energy of B-Mo complexes and stated that this co-segregation tendency enhances grain boundary cohesion [14]. Karlsson et al. [32] observed that grain boundary B levels were significantly higher in Mo-containing 316 steel compared to Mo-free steel, suggesting that Mo enhances B segregation. Mei et al. [33] corroborated these findings through first-principles calculations, showing that B and Mo exert the strongest boundary-strengthening effects among the 22 alloying elements investigated, counteracting the weakening effects of Zn on BCC iron grain boundaries. According to Straumal and Baretzky [34], the wetting of a GB in solid phase by a liquid phase is thermodynamically feasible when the energy of the GB is equal or higher than the two solid/liquid interfaces produced after wetting (σ_GB_ ≥ 2σ_SL_). According to Figure 9, they showed that lowering the grain boundary energy will result in a higher wetting temperature. Therefore, it can be concluded that, since Mo and B segregation on PAGBs reduces the GB energy, it results in the delayed wetting of GBs in the 0.2 Mo samples, which subsequently results in less LME susceptibility compared to the 0 Mo sample.

Figure 7a shows approximately 70 at% Zn within the LME cracks, attributed to Γ-Fe_3_Zn_7_ phase, which corresponds to Zn_liq_ at the welding temperature [35] and also 4 at% Cu, attributed to the copper electrode. Figure 7b indicates Si rejection toward the steel side of the liquid/solid interface, consistent with the limited solubility of Si in liquid Zn [36], and limited solubility of minor elements in liquid Zn, except for Mn. Figure 7c,d reveals the presence of Mo-rich carbides in the steel substrate, near the solid/liquid interface. This observation aligns well with that of Gupta et al. [37], who, based on CALPHAD calculations, identified stable M_7_C_3_ carbides at 630–670 °C. These particles were pre-existing in the microstructure since they were located in the base metal, which was not affected by the heat during RSW.

Figure 8 illustrates the diffusion behavior of Zn along and normal to the crack in the 0 Mo and 0.2 Mo alloys. The higher diffusion of Zn ahead of the LME crack (Figure 8b,f) along with Zn and Mo acting as ferrite stabilizers at high temperatures [38,39], facilitate the formation of grain boundary α-Fe(Zn) in the 0.2 Mo sample. Figure 8g,h shows there is also more lateral diffusion of Zn in the bulk of 0.2 Mo sample. Thus, diffusion along the grain boundary ahead of the crack and normal to the crack, into the bulk, leads to the consumption of the Zn and reduces the reservoir of liquid Zn feeding into the crack. It is reported that Zn and Mo compete over the segregation sites in the GBs [33]. Since Mo has a high atomic radius and a very low diffusion coefficient in austenite [40], this process will be sluggish, postponing the cracking of the grain boundary. Also, as mentioned earlier, Mo and B segregation on the PAGBs increases their cohesion strength [33]. So, more applied stress will be required to break Fe-Fe bonds in the grain boundaries, further delaying LME cracking.

## 5. Conclusions

In this study, the potential impacts of Mo addition on the LME susceptibility of AHSS were evaluated from different aspects. Below, the observations made here are summarized:

- RSW simulations of the shoulder region showed that the sample experienced temperatures higher than Ac_3_ during the welding. Comparing the RSW and Gleeble samples revealed that, in both cases, LME cracks propagated along PAGBs and their microhardness values were comparable (~545 ± 8 Hv). Thus, the austenization of the steel during RSW was implied.
- The larger force and stroke observed for the 0.2 Mo sample during hot tensile testing were accompanied by less LME cracking in the cross section of this sample in comparison to the 0 Mo alloy, without any significant difference in their grain sizes.
- SEM images showed that longer cracks, with more crack openings, were observed in the cross-section of the resistance spot-welded 0 Mo sample. This resulted in a higher crack severity index for the 0 Mo sample (0.61) in comparison to the 0.2 Mo sample (0.45).
- APT results shed light on the enhanced segregation of B on PAGBs in the presence of Mo, leading to increased cohesion of these grain boundaries and delayed LME cracking.
- STEM-EDS results showed a more gradual drop in Zn profile for the 0.2 Mo sample, as a result of more diffusion of Zn in the substrate, consuming the Zn reservoir for further feeding the liquid Zn inside the LME crack.

## Figures and Tables

**Table 1:** Chemical composition of the experimental alloys (wt%).

Steel	C	Si	Mn	Mo	Cr	B
0 Mo	0.2	1.5	2	0	0.41	0.0003
0.2 Mo	0.2	1.5	2	0.2	0.41	0.0003

**Figure A1:**
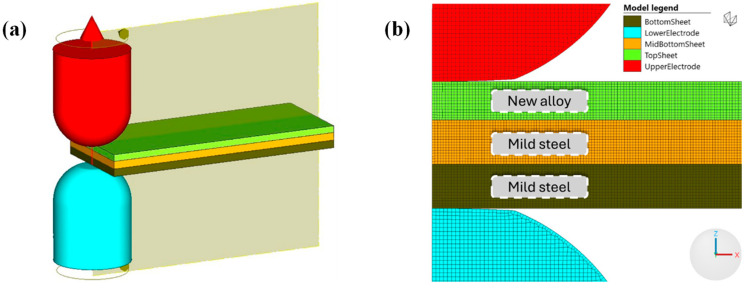
Representation of the RSW process showing (**a**) the chosen plane in the 3D mode and (**b**) zoom-in on the mesh and sheet sequence.

## Data Availability

The original contributions presented in this study are included in the article. Further inquiries can be directed to the corresponding author.

## Abbreviations

The following abbreviations are used in this manuscript:

PAGB	Prior Austenite Grain Boundary
GB	Grain Boundary
RSW	Resistance Spot Welding
LME	Liquid Metal Embrittlement
AHSS	Advanced High Strength Steel

## Appendix A

### Appendix A.1. Finite Element Simulation Model

The finite element model was built and simulated in Simufact. It consists of a sequentially coupled electro-thermo-mechanical simulation model that is widely reported in the literature [41,42,43,44]. Since this approach does not consider electromagnetic stirring effects such as molten metal convection, as in [45,46], the model requires initial calibration based on a known experimental nugget diameter. This calibration was carried out based on experimental data obtained for the newly developed alloy proposed in this work.

### Appendix A.2. Mesh and Boundary Conditions

Leveraging the axisymmetric nature of the process is an efficient way of simulating spot welding. Figure A1 shows the model setup and sequence of sheets.

The five geometries were meshed using 0.1 mm element size with element type 10. According to [47], Element Type 10 is a four-node, isoparametric, arbitrary quadrilateral written for axisymmetric applications. This element uses bilinear interpolation functions where strains tend to be constant throughout the element (plane strain). The stiffness of this element is formed using a four-point Gaussian integration and it should be used for nearly incompressible behavior. Its heat transfer element counterpart is Element 40, which electromagnetic models also support.

### Appendix A.3. Material Properties

The three sheets and two electrodes were modeled as deformable bodies with heat transfer capability. Electrodes were assigned CuCrZr, and the two bottom sheets were assigned mild steel properties, both of which are available in the software.

Data on the thermo-mechanical properties of the new steel as a function of temperature are not available in the available literature since this is a newly proposed alloy. Instead, this information was derived using JMatPro^®^ 11.0, a commercial software for material modeling. Furthermore, another commercial thermodynamics tool, Thermo-Calc^®^ 2023b, was employed to calculate properties such as bulk electrical resistivity across various temperatures, as well as the liquidus and solidus temperatures and the start temperature for martensite transformation.

The stress–strain curves generated by JMatPro were adjusted in the following way. Initially, a scaling factor was established by comparing the yield stress predicted by JMatPro at room temperature to the experimental results. A similar scaling factor was also determined for the ultimate tensile stress at room temperature. These two scaling factors are subsequently applied to the yield and tensile stress predictions from JMatPro at other temperatures, and the revised values are then entered into Simufact 2024.1.

### Appendix A.4. Contact Interactions

The interactions at the contact points in the RSW simulation exhibit significant nonlinearity, particularly regarding electrical and thermal contact properties. Equation (A1) illustrates the equation for gap thermal conductance utilized by Simufact. In this equation, the transfer of heat between two bodies depends on the contact pressure, as well as the thermal conductivity and yield stress of the materials. *P*_1_ through *P*_4_ represent numerical constants; *k*_1_ and *k*_2_ denote the thermal conductivity of body 1 and body 2, respectively; *F_n_* signifies the normal contact stress; and *σ_d_* indicates the yield stress of the softer material in the contacting pair. Both thermal conductivity and yield stress are expressed as functions of the local nodal temperature.

(A1) ht=P1k1+k221P21−e−P3Fnσd+P3P4

The electrical contact resistance utilized in the current model is based on [48]. It is described by Equation (A2), where *r*_0_ acts as a scaling factor, p_0_ denotes a reference pressure, *p_k_* is a corrective pressure to prevent unrealistic outcomes, *ε_p_* serves as a pressure exponent, *ε_p_* also represents a temperature exponent, and *T_lim_* is a temperature factor.

(A2) $$\rho \left(T,p\right)=r_{0}\cdot {\left(\frac{p-p_{k}}{p_{0}-p_{k}}\right)}^{\varepsilon_{p}}\cdot {\left(\frac{T-T_{lim}+\left(293-T\right)\cdot 2^{\frac{-1}{\varepsilon_{T}}}}{293-T_{lim}}\right)}^{\varepsilon_{T}}$$
